# *Salmonella* Typhi in the Democratic Republic of the Congo: Fluoroquinolone Decreased Susceptibility on the Rise

**DOI:** 10.1371/journal.pntd.0001921

**Published:** 2012-11-15

**Authors:** Octavie Lunguya, Veerle Lejon, Marie-France Phoba, Sophie Bertrand, Raymond Vanhoof, Jan Verhaegen, Anthony Marius Smith, Karen Helena Keddy, Jean-Jacques Muyembe-Tamfum, Jan Jacobs

**Affiliations:** 1 National Institute for Biomedical Research, Kinshasa, Democratic Republic of the Congo; 2 University Hospital of Kinshasa, Kinshasa, Democratic Republic of the Congo; 3 Department of Clinical Sciences, Institute of Tropical Medicine, Antwerp, Belgium; 4 Institute of Public Health, Brussels, Belgium; 5 University Hospital Leuven, Leuven, Belgium; 6 Centre for Enteric Diseases, National Institute for Communicable Diseases, National Health Laboratory Service, Johannesburg, South Africa and Faculty of Health Sciences, University of the Witwatersrand, Johannesburg, South Africa; Massachusetts General Hospital, United States of America

## Abstract

**Background:**

Drug resistance of *Salmonella enterica* serovar Typhi (*Salmonella* Typhi) to first-line antibiotics is emerging in Central Africa. Although increased use of fluoroquinolones is associated with spread of resistance, *Salmonella* Typhi with decreased ciprofloxacin susceptibility (DCS) has rarely been reported in Central Africa.

**Methodology/Principal Findings:**

As part of a microbiological surveillance study in the Democratic Republic of the Congo (DR Congo), *Salmonella* Typhi isolates from bloodstream infections were collected prospectively between 2007 and 2011. The genetic relationship of the S*almonella* Typhi isolates was assessed by pulsed-field gel electrophoresis (PFGE). The antimicrobial resistance profile of the isolates was determined and mutations associated with DCS were studied. In total, 201 *Salmonella* Typhi isolates were collected. More than half of the *Salmonella* Typhi isolates originated from children and young adults aged 5–19. Thirty different PFGE profiles were identified, with 72% of the isolates showing a single profile. Multidrug resistance, DCS and azithromycin resistance were 30.3%, 15.4% and 1.0%, respectively. DCS was associated with point mutations in the *gyrA* gene at codons 83 and 87.

**Conclusions/Significance:**

Our study describes the first report of widespread multidrug resistance and DCS among *Salmonella* Typhi isolates from DR Congo. Our findings highlight the need for increased microbiological diagnosis and surveillance in DR Congo, being a prerequisite for rational use of antimicrobials and the development of standard treatment guidelines.

## Author Summary

Typhoid fever, caused by infection with *Salmonella enterica* serovar Typhi (*Salmonella* Typhi), is an important health problem in sub-Saharan Africa. Multidrug resistance of *Salmonella* Typhi to the first line antibiotics is spreading and treatment of typhoid fever increasingly relies on fluoroquinolone antibiotics such as ciprofloxacin. Increased use of fluoroquinolones is however associated with spread of resistance as well. In sub-Saharan Africa, microbiological cultures to detect invasive bacterial diseases are frequently absent and the extent of the problem is poorly known. In the present study, 201 *Salmonella* Typhi isolates were collected between 2007 and 2011 in DR Congo, mainly from children and young adults. For the first time, widespread *Salmonella* Typhi multidrug resistance (30.3%) and decreased ciprofloxacin susceptibility (15.4%) is described in Central Africa. Decreased ciprofloxacin susceptibility was associated with point mutations in the quinolone resistance determining region of the *gyrA* gene. Resistance to azithromycin, an alternative for treatment of uncomplicated typhoid fever in the case of decreased ciprofloxacin susceptibility, was still rare (1.0%). Our findings demonstrate emergence of multidrug resistance and fluoroquinolone decreased susceptibility in DR Congo, and highlight the need for increased microbiological diagnosis and surveillance, being a prerequisite for rational use of antimicrobials and the development of standard treatment guidelines.

## Introduction

Typhoid fever is endemic in the Democratic Republic of the Congo (DR Congo). Although antimicrobial resistance data are sparse for Central Africa, drug resistance to first-line antibiotics is clearly emerging [1]. Also in DR Congo, multidrug resistance (MDR) [defined as co-resistance to first-line antibiotics ampicillin, chloramphenicol and trimethoprim/sulphamethoxazole (TMP-SMX)] in *Salmonella enterica* serotype Typhi (*Salmonella* Typhi) has been observed [2], [3]. Facing widespread MDR, fluoroquinolones have become the drugs of choice for treating typhoid fever, but their increased use has been associated with a spread in low-level fluoroquinolone resistance - further referred to as decreased ciprofloxacin susceptibility (DCS) [4]. *Salmonella* Typhi with DCS is common in Asia and has been reported in East Africa and South Africa [5]–[7], but apart from a single case reported from Cameroon [8], and two cases in DR Congo, *Salmonella* Typhi with DCS has not yet been observed in the central African region [2], [3], [9].

The present study describes the antimicrobial resistance profile of a prospective collection of *Salmonella* Typhi isolates recovered as part of a microbiological surveillance study from blood cultures obtained from patients in DR Congo over the years 2007–2011. Mutations associated with DCS were studied and the genetic relationship of the *Salmonella* Typhi isolates was assessed.

## Methods

### Ethics statement

Ethical approval was granted by the Ethical Committee of the University of Antwerp, Belgium and from the Ministry of Health in DR Congo. The present study complies with the World Health Organization and international guidelines (European Society of Clinical Microbiology and Infectious Diseases Study Group for Antimicrobial Resistance Surveillance and Clinical Laboratory Standards Institute) on antibiotic surveillance for which no recommendation for an informed consent has been issued. The diagnostic procedure – blood cultures – is part of the standard diagnostic work-up of patients with a suspicion of bacteremia. Clinical information -as presented- and information about use of antibiotics was the standard information present on the laboratory request form. Data have been reviewed and analyzed anonymously.

### Study setting, bacterial culture and identification

**Figure 1:**
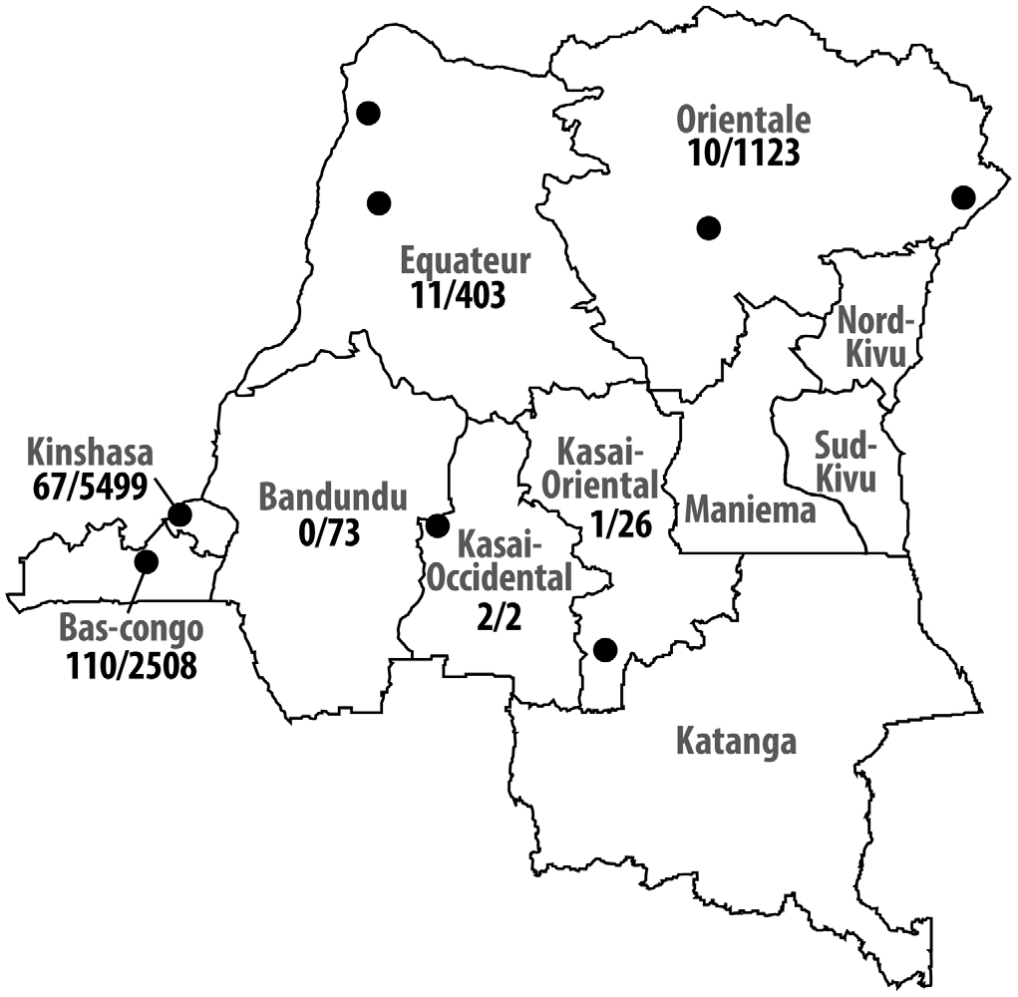
Origin of blood cultures and *Salmonella* Typhi isolates in DR Congo. Number of *Salmonella* Typhi grown/number of blood cultures received at INRB. •: Places where *Salmonella* Typhi positive cultures were obtained.

Between April 2007 and January 2011, blood cultures were performed on 9,634 patients suspected of typhoid fever or other systemic infections. Patients were seen at health care facilities in seven out of 11 provinces in DR Congo: Kinshasa, Bas-Congo, Bandundu, Equateur, Kasai Occidental, Kasai Oriental and Oriental province (Figure 1). In Kinshasa, health care facilities involved in the detection and study of the epidemic increase of typhoid fever-associated peritonitis of 2004 were selected [3]. Health care facilities in other provinces were recruited based on the past or actual existence of microbiological laboratories, professional contacts and the accessibility to reliable shipment facilities. Criteria for blood culture sampling were clinical suspicion of bacteremia associated with a local (pneumonia, urinary tract infection, meningitis or other) or systemic (typhoid fever, endocarditis) infection diagnosed at consultation or admission. Typhoid fever was defined according to the case definitions of the Ministry of Health surveillance of communicable diseases [10]. At the start of the surveillance project, teams of clinicians and laboratory technicians were trained in indications and sampling of blood cultures. For children <14 years, 1–4 ml of blood was inoculated into a pediatric blood culture vial (Bact/ALERT FP; bioMérieux; Marcy L'Etoile; France). For adults, 2×10 ml of blood was inoculated into aerobic blood culture vials (Bact/ALERT FA; bioMérieux; Marcy L'Etoile; France). Age, gender, geographic origin, use of antibiotics prior to blood culture sampling and presumptive diagnosis (focus of bacteremia including suspicion of typhoid fever) were recorded. Inoculated vials were shipped to the Institut National de Recherche Biomédicale (INRB) in Kinshasa, incubated at 35°C and daily checked for growth by visual inspection of the chromatographic growth indicator. Grown cultures were Gram stained, subcultured and identified to the species level. Skin or environmental bacteria (coagulase negative staphylococci, *Corynebacterium* spp., *Propionibacterium acnes* and *Bacillus* spp.) were categorized as contaminants, the other bacteria were considered as clinically significant organisms [11]. Suspected colonies of *Salmonella* were identified as *Salmonella* Typhi using standard biochemical methods (characteristic aspect on Kligler Iron Agar (acid from glucose, no gas, trace of H_2_S), negative tests for urease, oxidase, β-galactosidase and indole production tests, positive tests for lysine decarboxylase) and the serotype of *Salmonella* Typhi (O:9;H:d;Vi+) was confirmed with commercial antisera (Remel, Lenexa, Kansas). Identity of *Salmonella* species isolates was confirmed using the Vitek II system (Card GN21 341, bioMérieux). At the National Reference Laboratory for *Salmonella* and *Shigella* (Institute of Public Health, Brussels), the serotype of the *Salmonella* isolates was re-confirmed by slide agglutination with commercial monospecific antisera (Sifin, Berlin, Germany), following the Kauffmann-White scheme [12]. For analysis in the present study, only the first isolate per patient was considered.

### Antimicrobial susceptibility

Susceptibility tests for ampicillin, cefotaxime and TMP-SMX were performed using the Vitek II system (Card AB AST-N156, bioMérieux). For nalidixic acid, ciprofloxacin, chloramphenicol and azithromycin, minimal inhibitory concentration (MIC) values were determined using the E-test macromethod (bioMérieux). Breakpoints for resistance were respectively ≥32 mg/l for ampicillin, ≥4 mg/l for cefotaxime, ≥32 mg/l for naladixic acid, ≥16 mg/l chloramphenicol (considering intermediate susceptible isolates resistant) and ≥4/76 mg/l for TMP-SMX using Clinical and Laboratory Standards Institute definitions [13]. DCS was defined according to European Committee on Antimicrobial Susceptibility testing (EUCAST) V 2.0. guidelines, *i.e.* a MIC-value for ciprofloxacin >0.064 mg/l [14]. For azithromycin, EUCAST V.2.0. suggests MICs of >16 mg/l as defining resistance [14]. Multidrug resistance was defined as co-resistance to ampicillin, chloramphenicol and TMP-SMX.

Screening for mutations causing DCS was performed by amplification and sequencing of the quinolone resistance-determining regions (QRDRs) of the *gyrA*, *gyrB*, and *parC* genes. The presence of the plasmid-mediated quinolone resistance *qnr* genes (*qnrA*, *qnrB*, and *qnrS*) was determined using PCR [15].

### Pulsed-field gel electrophoresis

Pulsed-field gel electrophoresis (PFGE) was performed according to the PulseNet protocol for molecular subtyping of *Salmonella* [16], using *Xba*I as restriction enzyme (New England Biolabs, Leusden, The Netherlands). For cluster analysis, Bionumerics 5.1 was used (Applied Maths NV, Sint-Martens-Latem, Belgium), with as comparison settings the Dice similarity coefficient and UPMGA dendrogram type (optimization 0.50%, position tolerance 1.50%). PFGE profiles obtained were compared to PFGE of *Salmonella* Typhi profiles stored: In 1° the Institute of Public Health (Brussels) database, originating from Belgium (n = 27), Morocco (n = 8), Egypt (n = 1), Burkina Faso (n = 1), Niger (n = 1), Cambodia (n = 20), India (n = 11), Pakistan (n = 7), Bangladesh (n = 3), Indonesia (n = 1), Sri Lanka (n = 1), and Thailand (n = 1); 2° the PFGE database of the Centre for Enteric Diseases (CED) of the National Institute for Communicable Diseases in South Africa, containing patterns for 730 *Salmonella* Typhi isolates.

### Data analysis

All data were entered in an Excel database (Microsoft Corporation, Redmond, Washington, USA). Proportions were assessed for statistical significance using the Chi square test, considering *p*<0.05 as significant (Stata 10, StatCorp, Texas, USA).

## Results

From the 9634 blood cultures performed, 989 (10.3%) clinically significant organisms were grown, including 201 isolates of *Salmonella* Typhi, representing respectively 2.1% of all blood cultures and 20.3% of all clinically relevant organisms. There were no annual or seasonal differences in isolation rates. The geographic site of cases, positive on blood cultures for *Salmonella* Typhi in DR Congo, is shown in figure 1. Among the 201 *Salmonella* Typhi blood cultures isolates, 110 (54.7%) were recovered from Bas-Congo and 67 (33.3%) from Kinshasa. In Kinshasa, the isolation rate of *Salmonella* Typhi (67/5465, 1.2%) was lower compared to all other provinces in DR Congo (134/4064, 3.4%, *p*<0.001). The median age of patients infected with *Salmonella* Typhi was 15 years (interquartile range 8–25), but infection of young children was also common (Table 1). Over half of blood cultures were in children <10 years, yet 32.8% of the *Salmonella* Typhi isolates recovered were from children in this age group. The most affected age group were persons aged 10–19 years, in whom nearly 60% of the organisms isolated were *Salmonella* Typhi. In addition, this age group contributed to only approximately 10% of the blood culture samples, yet accounted for 30% of all *Salmonella* Typhi isolates recovered.

**Table 1:** Number of blood cultures, clinically significant organisms and *Salmonella* Typhi in function of age.

Age group (in years)	Blood cultures	CSO	*Salmonella* Typhi	*Salmonella* Typhi/CSO	*Salmonella* Typhi/total number *Salmonella* Typhi	*Salmonella* Typhi/blood cultures
0–4	3949	485	22	4.5%	10.9%	0.6%
5–9	1101	117	44	37.6%	21.9%	4.0%
10–19	1100	105	60	57.1%	30.3%	5.5%
20–29	1011	81	33	40.7%	16.4%	3.3%
30–39	823	59	20	33.9%	10.0%	2.4%
40–49	549	49	9	18.4%	4.5%	1.6%
≥50	894	69	13	18.8%	6.5%	1.5%
ND	207	24	0	0%	0%	0.0%
Total	9634	989	201	20.3%	100.0%	2.1%

CSO: Clinically significant organisms [*Salmonella,* other *Enterobacteriaceae (Klebsiella spp., Enterobacter spp*., *Citrobacter spp., Escherichia coli), Staphylococcus aureus, Candida sp., Streptococcus spp*.].

ND: not determined.

In patients with clinically significant organisms, presumptive diagnosis of typhoid fever was made in 53.0% (524/989) of the cases. In the 201 patients from whom *Salmonella* Typhi was cultured, presumptive diagnosis of typhoid fever at the moment of sampling was made in 80.6% (162/201) of the cases. A total of 21.0% (34/162) of these patients suffered from abdominal distension and/or gastro-intestinal bleeding and were classified as complicated typhoid fever. Other presumptive diagnoses (for several patients more than one presumptive diagnosis was mentioned) included complicated urinary tract infection (14.4%), pneumonia (7.0%), meningitis (2.0%), malaria (5%) and other non-specified causes of bacteremia (16.4%); for three patients (1.5%), no data were available. Nearly half (93/201, 46.3%) of the patients had received antibiotics within 48 hours prior to sampling of blood cultures, mostly first-line antibiotics.

Resistance against ampicillin, chloramphenicol or TMP-SMX were observed in 64.7% (130/201), 41.3% (83/201) and 57.7% (116/201) of isolates, respectively. MDR and DCS were observed in 30.3% (61/201) and 15.4% (31/201) of isolates respectively; combined MDR and DCS occurred in 7.5% (15/201) of isolates. Isolates with DCS corresponded to nalidixic acid resistant isolates and vice versa. Only two (1.0%) isolates had azithromycin MIC values exceeding 16 mg/l (i.e. 24 mg/l); both isolates were also resistant against ampicillin and TMP-SMX (one also combined with DCS and nalidixic acid resistance). No cefotaxime resistance was observed. Fifty-one isolates (25.4%) were fully susceptible to all three first-line antibiotics and 50 (24.9%) were susceptible to all seven antibiotics tested. There was no apparent relationship between antimicrobial resistance and patient age, year of isolation, province of isolation or administration of antibiotics prior to isolation.

All 31 isolates with DCS were analysed for mutations in the QRDRs of the *gyrA*, *gyrB*, and *parC* genes. No mutations were detected in *gyrB* or *parC* genes. No *qnrA* or *qnrB* genes were detected. For one isolate, the *qnrS* gene was detected. Apart from a mutation at codon 133 in the *gyrA* gene (conferring a Glu to Gly change in the GyrA protein), which was also present in nalidixic acid susceptible strains, all 31 isolates had DCS-associated mutations in the *gyrA* gene, conferring the following amino acid mutations in the GyrA protein:(i) Ser83 changed into Phe or Tyr (n = 22), or (ii) Asp87 changed into Gly, Tyr or Asn (n = 9).

Among 185 isolates tested for PFGE, 30 different profiles were observed (Supplemental file). In 132/185 isolates (71.4%), an indistinguishable PFGE profile occurred. This profile was the main or single PFGE profile over time and geography, although its proportion was lower (*p* = 0.02) in Kinshasa province (39/64 isolates, 60.9%) compared to the other provinces in DR Congo (93/121, 76.9%). In Kinshasa, the highest variation of profiles - in total 22 - was noted. Of the isolates showing the main/predominant profile, 31.1% (41/132) were MDR and 17.4% (23/132) were DCS, while 11 (8.3%) showed a combined MDR and DCS. Comparison of the PFGE profiles of *Salmonella* Typhi from DR Congo with isolates from other geographic origins revealed one PFGE profile from a *Salmonella* Typhi isolate from DR Congo that was indistinguishable from a PFGE profile from an isolate recovered in Belgium, and 5 Congolese PFGE profiles that were indistinguishable from PFGE profiles of 9 *Salmonella* Typhi isolates recovered in South Africa.

## Discussion

The present study demonstrated widespread MDR and DCS among *Salmonella* Typhi isolates from DR Congo. DCS resistance was associated with point mutations in the *gyrA* gene. Azithromycin resistance was rare. Nearly 75% of isolates were associated with a single PFGE profile. Our data indicate that infection with *Salmonella* Typhi is highly clinically relevant for school-aged children and young adults.

Our study has some limitations. Despite our attempt to survey large parts of DR Congo, the geographical origin of the isolates could have been biased by logistic difficulties. Connections and communications by road are very limited in DR Congo and except from the Bas-Congo and Kinshasa provinces, samples had to be shipped and transported by air plane. Apart from shipment delays, antibiotic use prior to sampling may have affected culture yields and biased the resistance profiling towards resistant organisms. Further, our study did not allow calculation of incidence rates because referral patterns and catchment populations among participating centers differed; for instance financial constraints prevent many patients from consulting. Medical doctors are not familiar with microbiological culture tools; they rely on the clinical picture or the Widal test for diagnosis of typhoid fever [10]. There were undoubtedly differences in application and interpretation of the clinical criteria as well variations in sampling intensity. Drug efflux was not examined in DCS strains, so we cannot exclude the possibility that it played a role in the observed fluoroquinolone resistance. Finally, PFGE as a genotyping method has several limitations, including insensitivity when dealing with likely clonally related strains. As a consequence, single nucleotide polymorphism differences that may be important in identifying subtypes among closely related *Salmonella* Typhi strains may have been missed [17]. On the other hand, despite these limitations, the yield of clinically significant organisms was in line with international standards and similar for all settings and over time. In addition, we were able to document antimicrobial resistance patterns at a large scale in the central African region where the typhoid fever situation is notoriously poorly characterized [4].

*Salmonella* Typhi infection appears to prevail among school-aged children and young adults. Of note, in the present study, *Salmonella* Typhi was also isolated from children under 10 years of age at a positivity rate of 32.8%. This value is higher than previously reported from the Kivu province (not included in our current study) in Eastern DR Congo where a previous study reported a positivity rate of 7.1% amongst children of this age group, amongst a total of 409 *Salmonella* Typhi cultures sampled during the years 2002–2006) [2]. Patients' age distribution in the present study compares with that observed in countries with medium to high incidence rates of typhoid fever [18], and was similar to a Ugandan report [9] although different from data obtained in South Africa and Malawi, where young children seem relatively less affected [5], [19]. This has been highlighted by Bhutta and contrasted with south Asian countries [20], although some geographic differences may exist [19]. In urban Kinshasa, the isolation rate of *Salmonella* Typhi appeared to be lower compared to the other (more rural) provinces in DR Congo, which seems in contrast to observations in Kenya [21]. Although this seems to suggest that incidence might be lower in urban than in rural areas, one should be careful interpreting these results since referral patterns and catchment populations may have differed, as well as the type of sampling sites. In the provinces, sampling sites were uniquely referral hospitals, whereas in Kinshasa, private clinics were also included. Suspicion of meningitis in a minority of patients, suggests that neurological symptoms might have been present [5].

Although commonly described in our present study, MDR to first line antibiotics, was less prevalent in the current isolate collection compared to previous reports of MDR in *Salmonella* Typhi from DR Congo [2], [3], for Nigeria [22], Kenya [6], [21], and Malawi and Mozambique [5]. Although discriminative power of PFGE using one restriction enzyme might have been insufficient [23], PFGE suggests limited genotypic diversity with a single *Salmonella* Typhi clone prevailing in DR Congo, while in Kinshasa a more diverse panel of profiles was observed. As observed in Kenya, MDR and sensitive clones seem to share similar PFGE patterns [6]. The *Salmonella* Typhi PFGE patterns from DR Congo have been submitted to the Global PulseNet *Salmonella* Typhi Database (www.pulsenetinternational.org), for consideration to be included in to the database in order to allow for international comparison of the *Salmonella* Typhi isolates. A limited comparison of the Congolese PFGE patterns with patterns of *Salmonella* Typhi from other origins revealed six patterns that were indistinguishable from Belgian and South African PFGE patterns. Exchange of *Salmonella* isolates between Belgium and DR Congo seems plausible in view of the historical links and the frequent travelling between the two countries. It was also not unexpected to find DR Congo pulsotypes indistinguishable from South African pulsotypes, as there is much migration of DR Congo nationals into South Africa.

For the first time in Central-Africa, widespread DCS related to the presence of *gyrA* mutations was reported. DCS may lead to treatment failure [24] and points to emerging fluoroquinolone resistance. The DCS rate was similar to the rate described in Nigeria, with comparable MIC50 and MIC90 values [22]. It appeared higher than in Kenya [6], [21], Malawi and Mozambique [5], South-Africa [7] and Uganda [9] although these studies might have applied older CLSI guidelines. Fluoroquinolone resistant isolates showed mutations in *gyrA* which conferred amino acid mutation at codons 83 and 87 in the GyrA protein; these mutations were similar to previous observations in South Africa [7]. The fact that DCS was invariably predicted by nalidixic acid resistance should be further explored; in particular future studies should be carried out to examine the predictive value of screening for DCS by simple nalidixic acid disk diffusion test [25]. As there is limited human migration between provinces (some of the presently studied provinces are only accessible by air or river), the similar rates of DCS over all provinces may suggest spontaneous and independent mutations induced by selection pressure rather than a single resistant clone spreading throughout the country. Indeed, fluoroquinolone antibiotics have been increasingly used in DR Congo since the MDR *Salmonella* Typhi outbreaks in 2004 [3]. Azithromycin may be a valuable alternative for treatment of uncomplicated typhoid fever in the case of DCS [4], and there were low levels of resistance to this antibiotic. However, as its patent has expired recently, it can be expected that the market in resource limited settings will be flooded by newer cheaper generics, with the danger of indiscriminate use resulting in the emergence and spread of azithromycin resistance. Indeed, from site visits we observed that azithromycin is now increasingly been introduced and promoted as an oral antibiotic for many indications.

The present results provide an early warning sign for the emerging resistance of a bacterial key-pathogen to affordable antibiotics in DR Congo. Together with recent data we described about the occurrence of MDR bacteria in drinking water in Kinshasa city [26], they provide evidence of a serious problem of antibiotic resistance in the community setting of DR Congo. The need for microbiological diagnosis and surveillance is highlighted. Surveillance not only timely detects outbreaks but is also a prerequisite for rational use of antimicrobials and the development of standard treatment guidelines [4], [27] which in turn are needed to contain antibiotic resistance.

### Transparency declaration

This study was funded by Directorate General of Development Cooperation of the Belgian Government through Institutional Collaboration INRB-ITM (Network Program on Laboratory Quality Management; Project 3.21). The funders had no role in study design, data collection and analysis, decision to publish, or preparation of the manuscript. The authors declare that they have no conflicting interests in relation to this work.

## Supporting Information

File S1 **Pulsed-field gel electrophoresis (PFGE) dendrogram of** ***Xba*** **I profiles of 185** ***Salmonella*** **Typhi isolates from DR Congo.** For each profile the total number of isolates and the number and percentage of resistant isolates for different antibiotics is given. Similarity between PFGE patterns was assessed by cluster analysis (Dice coefficient and UPGMA, tolerance and optimization of band position set at 1.5% and 0.5%). ^B, SA^ PFGE profiles observed in *Salmonella* Typhi isolates from respectively Belgium or South Africa. The number of corresponding isolates per profile is indicated. Amp: ampicillin. Chl: Chloramphenicol. TMP-SMX: trimethoprim/sulphamethoxazole. MDR: multidrug resistance. DCS: decreased ciprofloxacin susceptibility.
(TIF)
