# Supplementary figures and images for: Salmonella Typhi in the Democratic Republic of the Congo: Fluoroquinolone Decreased Susceptibility on the Rise
Source: PLoS Negl Trop Dis. 2012 Nov 15;6(11):e1921. doi: 10.1371/journal.pntd.0001921 (PMC3499407; doi:10.1371/journal.pntd.0001921)

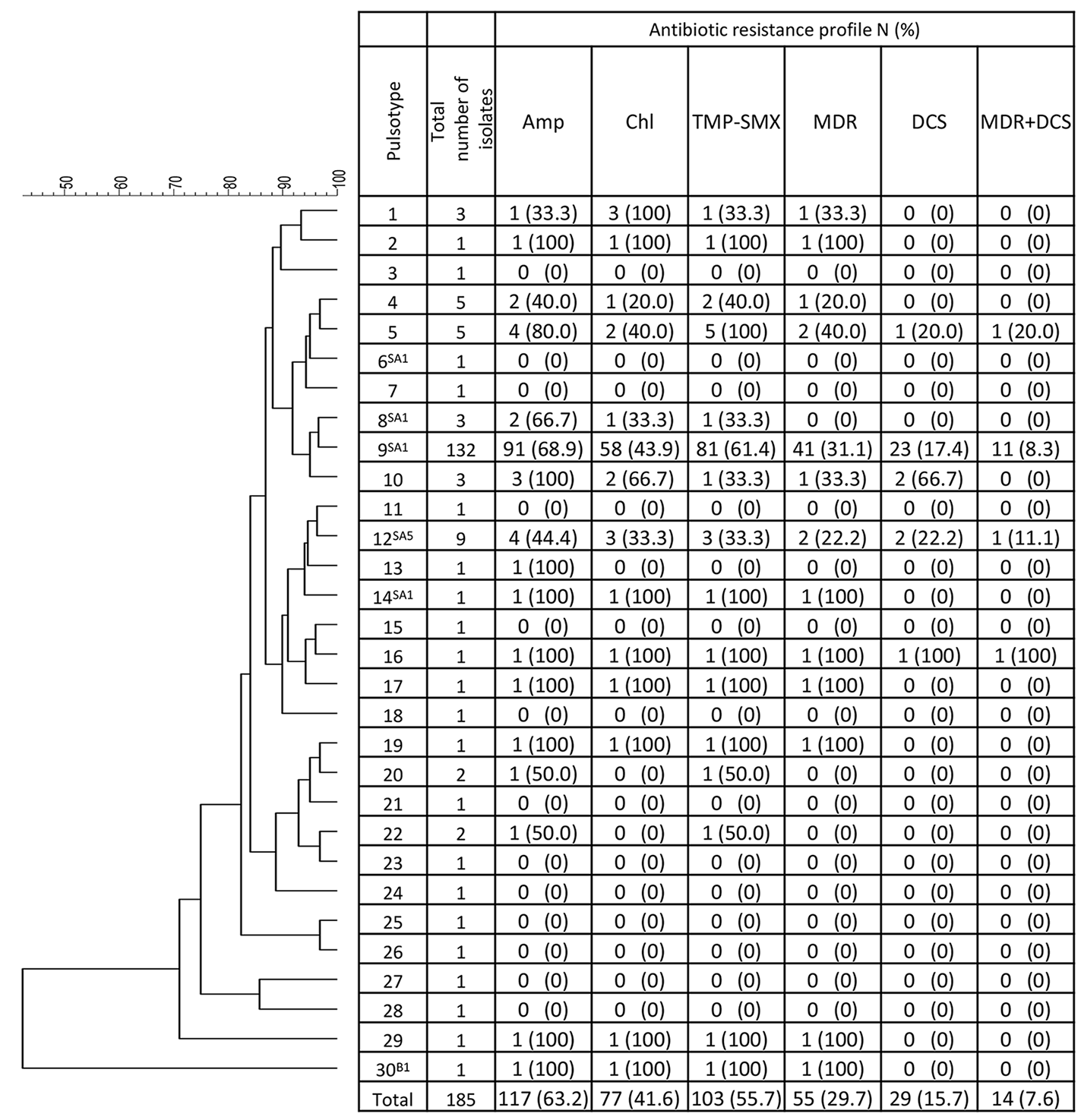

Supplement: File S1 — Pulsed-field gel electrophoresis (PFGE) dendrogram of Xba I profiles of 185 Salmonella Typhi isolates from DR Congo. For each profile the total number of isolates and the number and percentage of resistant isolates for different antibiotics is given. Similarity between PFGE patterns was assessed by cluster analysis (Dice coefficient and UPGMA, tolerance and optimization of band position set at 1.5% and 0.5%). B, SA PFGE profiles observed in Salmonella Typhi isolates from respectively Belgium or South Africa. The number of corresponding isolates per profile is indicated. Amp: ampicillin. Chl: Chloramphenicol. TMP-SMX: trimethoprim/sulphamethoxazole. MDR: multidrug resistance. DCS: decreased ciprofloxacin susceptibility. (TIF) [file pntd.0001921.s001.tif]
